# The ratio of measured to estimated glomerular filtration rate may be a marker of early mortality and dialysis requirement

**DOI:** 10.1186/s12882-021-02561-1

**Published:** 2021-11-07

**Authors:** James G. Heaf, Rafal Yahya, Morten Dahl

**Affiliations:** Zealand University Hospital, Roskilde, Denmark

**Keywords:** Uraemia, Glomerular filtration rate, dialysis

## Abstract

**Background:**

It has been suggested that, in patients with CKD stage 5, measured GFR (mGFR), defined as the mean of urea and creatinine clearance, as measured by a 24-h urine collection, is a better measure of renal function than estimated GFR (eGFR), based on the CKD-EPI formula. This could be due to reduced muscle mass in this group. Its use is recommended in the ERBP guidelines. Unplanned dialysis initiation (DI) is associated with increased morbidity, mortality, and reduced modality choice and is generally considered undesirable. We hypothesized that the ratio mGFR/eGFR (M/E) aids prediction of death and DI.

**Methods:**

All 24-h measurements of urea and creatinine excretion were extracted from the clinical biochemistry databases in Zealand. Data concerning renal diagnosis, comorbidity, biochemistry, medical treatment, mortality and date of DI, were extracted from patient notes, the National Patient Registry and the Danish Nephrology Registry. Patients were included if their eGFR was < 30 ml/min/1.73m^2^. The last available value for each patient was included. Follow-up was 12 months.

**Results:**

One thousand two hundred sixty-five patients were included. M/E was median 0.91 ± 0.43. It was highly correlated to previous determinations. It was negatively correlated to eGFR, comorbidity, high age and female sex. It was positively related to albumin and negatively to C-reactive protein. M/E was higher in patients treated with ACE inhibitors and diuretics but no other treatment groups.

On a multivariate analysis, M/E was negatively correlated with mortality and combined mortality/DI, but not DI. A post hoc analysis showed a negative correlation to DI at 3 months. For patients with an eGFR 10–15 ml/min/1.73m^2^, combined mortality and DI at 3 months was for low M/E (< 0.75) 36%, medium (0.75–1.25) 20%, high (> 1.25) 8%. A low M/E predicted increased need for unplanned DI.

A supplementary analysis in 519 patients where body surface area values were available, allowing BSA-corrected M/E to be analyzed, revealed similar results.

**Conclusion:**

A low mGFR/eGFR ratio is associated with comorbidity, malnutrition, and inflammation. It is a marker of early DI, mortality, and unplanned dialysis initiation, independently of eGFR, age and comorbidity. Particular attention paid to patients with a low M/E may lower the incidence of unplanned dialysis requirement.

**Supplementary Information:**

The online version contains supplementary material available at 10.1186/s12882-021-02561-1.

## Introduction

Exogenous clearance, as measured by radioactive isotopes, iohexol or iothalamate, is the gold standard of measuring GFR. These methods are cumbersome and expensive, and have generally been replaced in clinical practice by the Chronic Kidney Disease Epidemiology Collaboration (CKD-EPI) equation for estimated glomerular filtration (eGFR) [1, 2]. This is based on age, sex, p-creatinine and race. The accuracy of the equation is low (confidence interval ± 30%). In recent years, the use of the equation in patients with CKD stage 5 (CKD5) (eGFR< 15 ml/min/1.73m^2^) has been questioned. Firstly, the equation is inaccurate when compared to gold standard measurements, overestimating GFR by 1–3 ml/min/1.73m^2^ with a confidence interval of ±10 ml/min/1.73m^2^ [3–5]. Secondly, a number of large epidemiological studies [6–8] have showed a paradoxical inverse relationship between eGFR at dialysis initiation (DI) and subsequent survival: the higher the eGFR, the higher the mortality. There are two possible explanations.

1. Patients with high comorbidity start dialysis earlier, due to non-specific symptoms ascribed to uraemia. In particular, patients with heart failure or diabetes will have difficulty controlling fluid balance. Patients with a low p-albumin caused by chronic disease and inflammation may erroneously be assumed to be malnourished. Lasalle et al. [8] found early starters to be older, with more cardiac disease, diabetes and malnutrition. Others report similar findings [9–14]. However, correction for comorbidity fails to completely remove the association [9, 10, 14].
2. CKD5 is characterized by a reduction in muscle mass and protein intake, with a consequent reduction in creatinine production. This will result in a misleading increase in eGFR. Thus, while creatinine clearance is greater than eGFR among well-nourished CKD5 patients, it is lower if the patient is malnourished [15]. Urine-based measurements would a priori be expected to solve this problem. Urea excretion is a marker of protein intake, while creatinine excretion is a marker of muscular mass and overall nutritional status. Creatinine clearance is higher than true GFR due to tubular secretion, while urea clearance is lower due to tubular absorption. The measured GFR (mGFR), defined as the average of urea and creatinine clearance based on a 24-h urine collection, has been validated [16]. For CKD3 & 4 patients, the mGFR underestimates true GFR by 10%. After correction for this, the equation is as accurate as eGFR. Grootenhorst et al. [17] found that eGFR, but not mGFR, was negatively correlated to muscle mass. If mGFR was used instead of eGFR, the paradoxical association to mortality disappeared. The same result was seen in another study, where creatinine clearance was used to measure renal function [18]. In the absence of exogenous clearance studies, mGFR seems therefore preferable to eGFR in patients with CKD5. For this reason, the European Renal Best Practice guidelines recommend mGFR as the preferred method of GFR estimation in CKD5 [19].

Unplanned dialysis initiation (DI) is associated with increased morbidity and mortality [20–24]. Since the association is believed to be causal, physicians generally try to avoid it by planning DI in a timely manner. The prescription of maintenance dialysis is usually based on clinical problems combined with current eGFR. We hypothesized that a measurement of the ratio of mGFR to eGFR (M/E) would provide supplementary information, independent of eGFR, in predicting requirement for dialysis.

## Methods

All available 24-h measurements of urea and creatinine excretion were extracted from the clinical biochemistry databases in Zealand, Denmark. In addition, measurements were extracted from the departmental uraemia database (Nefreg). Since electronic registration was only available after 2009, 91% of values were after this date. Simultaneous plasma values of creatinine, urea, albumin and C-reactive protein were noted. The eGFR, and the mGFR uncorrected for body surface area, were calculated. Patients were included if at least one eGFR value was < 30 ml/min/1.73m^2^. The ratio of mGFR to eGFR (M/E) was calculated.

$$mGFR=\frac{24\ hour\ urine\ volume}{1.44\times 2}\times \left(\frac{Urine\ urea\ concentration}{Plasma\ urea\ concentration}+\frac{Urine\ creatinine\ concentration}{Plasma\ creatinine\ concentration}\right)$$

1.44 is the conversion factor for altering units from L/day to ml/minute

Data concerning renal diagnosis, comorbidity, mortality, and date of DI were extracted from the National Patient Registry and the Danish Nephrology Registry. The following comorbidities at each M/E measurement were registered: previous myocardial infarction, heart failure, other cardiac disease, cerebrovascular disease, peripheral atherosclerosis, pulmonary disease, hepatic disease, cancer and diabetes mellitus.

The circumstances surrounding the first dialysis were extracted from the DNR. DI was defined as planned if the access was an arteriovenous fistula or graft, a tunneled catheter as the patient’s planned permanent access, or a peritoneal dialysis catheter placed > 6 days before DI. Otherwise, DI was unplanned.

The last available value for each patient was included in the primary analysis. In GFR subgroup analyses the final value in each eGFR subgroup was used. For patients with repeat mGFR determinations, the reproducibility of M/E was investigated using initial values.

Weight, height, and body surface area (BSA) were extracted from patient notes. Since these data were only available in a minority of cases, uncorrected mGFR values were used in the primary analysis, and corrected values (mGFR_BSA_, M/E_BSA_) in a subgroup analysis.

Details of current medical treatment for a subgroup of patients attending a nephrology clinic were extracted from the patient notes in order to assess the effect of current medical treatment on M/E.

Unplanned DI was defined as primary dialysis access via a non-tunneled catheter, a temporary tunneled catheter or peritoneal dialysis treatment < 6 days after catheter placement.

Permission for the study was obtained from the Danish Data Protection Agency (REG-117-2018) and the Danish Patient Safety Authority (3–3013-2728/1). All methods were carried out in accordance with relevant guidelines and regulations.

### Statistics

Data are presented as mean ± standard deviation (SD) for normally distributed variables, median (interquartile range, IQR) for non-normally distributed variables, or numbers (percentage). Normally distributed variables were compared using the Students t-test and MANOVA. Non-normally distributed variables were compared using Mann-Whitney and categorical analysis using Chi square.

Kaplan Meier and Cox proportional hazards analysis were performed to identify the independent associations of eGFR and mGFR with DI and mortality. Since the stability of M/E over a long period was unknown, the patients were censored at 1 year after last M/E measurement. M/E was adjusted for eGFR and BSA (Model 1) and eGFR, BSA, age, sex and comorbidity (Model 2). A subgroup analysis of patients with an eGFR 10–15 ml/min/1.73^2^ was performed, as accurate prediction of DI is particularly relevant for this group. Mean substitution was used for missing BSA values. A post hoc analysis showed different survival results for the first 3 months, so a supplementary analysis for this period was performed.

A two-tailed probability level of < 0.05 was considered significant. Significance values were expressed as *p* < 0.05, *p* < 0.01, *p* < 0.001.

## Results

One thousand two hundred sixty-five patients were included. 626 had multiple measurements. Body surface area (BSA) was available in 519 patients, and their corrected ratio (M/E_BSA_) could be calculated. Patient clinical and biochemical details, and their relationship to M/E are shown in Table 1. The relationship of M/E to nutritional variables is shown in Table 2. Even after adjusting for body weight or BSA, M/E was significantly correlated to nutritional/inflammation variables, albumin and C-reactive protein in particular.

**Table 1:** Patient clinical and biochemical details at final M/E measurement, and their relationship to M/E. All patients and patients with BSA measurements

	All			With BSA		
		M/E			M/E	
		Absent	Present		Absent	Present
Number	1265			519		
Age (yrs)	68.0 ± 14.0			65.6 ± 13.9		
Age > 70 yrs	664 (52.4)	0.97 ± 0.47	0.86 ± 0.38^c^	234 (45.1)	0.92 ± 0.37	0.85 ± 0.30^a^
Sex (female)	465 (36.8)	0.96 ± 0.45	0.84 ± 0.39^c^	710 (36.8)	1.05 ± 0.43	0.92 ± 0.37
**Renal disease**						
Glomerulonephritis	172 (13.6)		1.02 ± 0.45	92 (17.7)		1.12 ± 0.44
Chronic interstitial	190 (15.0)		0.95 ± 0.44	80 (15.4)		0.95 ± 0.46
Polycystic	54 (4.3)		1.03 ± 0.40	35 (6.7)		1.11 ± 0.42
Diabetic	375 (29.6)		0.88 ± 0.43	158 (30.4)		0.96 ± 0.35
Hypertensive	101 (8.0)		0.94 ± 0.36	50 (9.6)		0.98 ± 0.33
ANCA vasculitis	53 (4.2)		0.88 ± 0.40	22 (4.2)		0.84 ± 0.31
Other	51 (4.0)		0.86 ± 0.44	24 (4.6)		1.01 ± 0.58
Unknown	176 (13.9		0.86 ± 0.39	40 (7.7)		0.95 ± 0.32
Not stated	93 (7.4)		0.86 ± 0.47	18 (3.5)		1.04 ± 0.57
**Comorbidity**						
Myocardial infarction	215 (17.0)	0.92 ± 0.43	0.89 ± 0.43	96 (18.5)	0.99 ± 0.41	1.01 ± 0.41
Heart failure	285 (22.5)	0.94 ± 0.44	0.80 ± 0.37^c^	105 (20.2	1.03 ± 0.42	0.90 ± 0.37^b^
Other heart	575 (45.5)	0.90 ± 0.44	0.93 ± 0.42	252 (48.6)	1.02 ± 0.42	0.98 ± 0.41
Cerebrovascular	200 (15.8)	0.93 ± 0.44	0.84 ± 0.38^b^	77 (14.8)	1.02 ± 0.42	0.89 ± 0.36^b^
Peripheral atherosclerosis	211 (16.7)	0.93 ± 0.44	0.84 ± 0.39^b^	83 (16.0)	1.01 ± 0.42	0.93 ± 0.34
Pulmonary	230 (18.2)	0.94 ± 0.44	0.80 ± 0.37^c^	78 (15.0)	1.02 ± 0.41	0.87 ± 0.38^b^
Hepatic	47 (3.7)	0.92 ± 0.43	0.67 ± 0.41^c^	17 (3.3)	1.00 ± 0.41	0.80 ± 0.44^a^
Cancer	270 (21.3)	0.92 ± 0.44	0.90 ± 0.37	109 (21.0)	1.02 ± 0.43	0.94 ± 0.33
Diabetes mellitus	550 (43.5)	0.92 ± 0.44	0.91 ± 0.44	252 (48.6)	1.01 ± 0.43	0.99 ± 0.39
**Plasma Biochemistry**		**R**			**R**	
Urea (mM)	22.2 ± 9.3	0.01		22.8 ± 8.8	0.09^a^	
Creatinine (mM)	338 ± 157	0.17^c^		268 ± 166	0.27^c^	
Potassium (mM)	4.1 ± 0.6	0.06		4.2 ± 0.5	0.02	
Sodium (mM)	139 ± 4	0.14^c^		138 ± 4	0.15^b^	
Bicarbonate (mM)	23.4 ± 4.7	−0.19^c^		22.8 ± 4.1	−0.07	
Calcium-ion (mM)	1.17 ± 0.11	0.12^b^		1.18 ± 0.11	0.14	
Phosphate (mM)	1.33 ± 0.45	−0.05		1.36 ± 0.44	0.00	
Albumin (g/L)	31.6 ± 6.6	0.24^c^		33.0 ± 5.5	0.15^b^	
C-reactive protein (mg/L)	10 (3–40)	−0.22^c#^		5 (3–18)	− 0.12^a^	
**Urine biochemistry**						
Protein (g/d)	0.9 (0.4–2.4)	0.28^c^		1.3 (0.5–3.1)	0.26^c^	
Creatinine (mM/d)	8.6 ± 4.4			9.2 ± 4.0		
Urea (mM/d)	272 (161–351)			270 (185–367)		
Clearance*						
eGFR	16.9 ± 7.1	−0.22^c^		15.6 ± 6.9	−0.25^c^	
Creatinine clearance	20.6 ± 12.7	0.42^c^		17.8 ± 9.7	0.37^c^	
Urea clearance	9.5 ± 6.3	0.59^c^		8.6 ± 5.3	0.57^c^	
Ratio urea to creatinine clearance	0.49 ± 0.23	0.18^c^			0.21^c^	
mGFR	15.1 ± 9.1	0.51^c^		13.2 ± 7.0	0.47^c^	
Difference mGFR to eGFR	−1.8 ± 7.3			−2.4 ± 5.6		
M/E	0,.91 ± 0.43			0.88 ± 0.34		

^a^:*p* < 0.05; ^b^:*p* < 001;^c^:*p* < 0.001. R: correlation coefficient. * ml/min or ml/min/1.73m^2^. ^#^: logarithmic transformation

**Table 2:** Relationship of M/E to nutritional variables

	All patients^**2**^			eGFR 10–15 ml/min/1.73m^**2**^		
	M/E			M/E		
	< 0.75	0.75–1.25	> 1.25	< 0.75	0.75–1.25	> 1.25
eGFR (ml/min/1.73m^2^)	17.4 ± 7.2	17.5 ± 7.0	14.3 ± 6.5^c^	12.5 ± 1.5	12.4 ± 1.5	12.2 ± 1.5
Urea excretion (mM/d)	108 ± 53	134 ± 45	151 ± 63^c^	95 ± 47	126 ± 43	142 ± 58^c^
Urea excretion/weight (mM/d/kg)	2.4 ± 1.5	3.7 ± 1.5	5.1 ± 2.9^c^	2.2 ± 1.3	3.6 ± 1.4	5.0 ± 2.9^c^
Creatinine excretion (mM/d)	4.0 ± 2.6	4.4 ± 1.9	5.0 ± 1.7^c^	3.8 ± 2.9	4.4 ± 1.7	5.0 ± 2.0^c^
Creatinine excretion/BSA (mM/d/m^2^)	2.8 ± 0.9	4.7 ± 1.0	6.6 ± 1.8^c^	2.5 ± 1.0	4.7 ± 0.9	6.7 ± 1.4^c^
C-reactive protein (mg/L)*	19 (6–56)	8 (3–27)	6 (3–18)^c^	32 (9–68)	9 (3–30)	7 (3–19)^c^
Albumin (g/L)	30 ± 7	33 ± 6	33 ± 6^c^	29 ± 7	32 ± 6	32 ± 7^c^

*Median (interquartile range). ^c^:*p* < 0.001 (group analysis)

The final M/E determination was highly (*p* < 0.001) correlated to the first, regardless of interval (1–12 months 233 patients, r = 0.68; > 12 months 98 patients, r = 0.68). For patients observed for more than 12 months, the M/E was unchanged (first value 1.09 ± 0.40, last 1.03 ± 0.41). mGFR was correlated to BSA (mGFR (ml/min) = − 4.7 + 10.1 x BSA (kg/m^2^), *p* < 0.001). The last M/E for patients with single and multiple determinations was similar (single 0.93 ± 0.43, multiple 0.90 ± 0.43).

M/E was negatively correlated to eGFR. the subgroup of patients with BSA measurements did not differ substantially from the group as a whole and the correlations to eGFR were similar. Both were lower than eGFR. They were lower in older, female and comorbid patients. They were related to albumin and C-reactive protein and biochemical markers of uraemia. Medical treatment data was available in 137 patients (Table 3). M/E was significantly higher in patients treated with ACE inhibitors and diuretics, but not to other treatment groups.

**Table 3:** Relation of medical treatment to M/E at last M/E measurement

		M/E	
		Not treated	Treated
Number	137		
eGFR	13.4 ± 5.1		
Antithrombotic	57 (41.6)	1.04 ± 0.39	1.05 ± 0.39
ACE inhibitor	21 (15.3)	1.01 ± 0.36	1.21 ± 0.50^a^
Angiotensin 2 antagonist	24 (17.5)	1.04 ± 0.41	1.04 ± 0.32
β-blocker	84 (61.3)	0.99 ± 0.35	1.07 ± 0.41
Calcium antagonist	55 (40.1)	0.99 ± 0.37	1.11 ± 0.42
Any antihypertensive	118 (86.1)	0.85 ± 0.32	1.07 ± 0.39^a^
Diuretics	95 (69.3)	0.93 ± 0.35	1.09 ± 0.40^a^
Sodium bicarbonate	53 (38.7)	1.03 ± 0.36	1.06 ± 0.44
ESA	51 (37.2)	1.04 ± 0.40	1.04 ± 0.38
Active vitamin D	51 (37.2	1.00 ± 0.35	1.10 ± 0.45

^a^: *p* < 0.05. ACE: angiotensin converting enzyme; ESA: erythropoiesis stimulating agent

M/E was negatively associated at one year (Table 4) with mortality and combined mortality/DI, but not DI alone, independently of eGFR and BSA. The association remained after adjustment for age, sex and morbidity. The results were similar for the subgroup of patients with an eGFR of 10–15 ml/min/1.73m^2^. The M/E had no predictive value for death or dialysis requirement if the eGFR was < 10 ml/min/1.73m^2^).

**Table 4:** Hazard ratios (%) for outcomes three and twelve months after last M/E measurement. Model 1: eGFR and M/E only; Model 2: adjusted for age, sex and comorbidity

		Model 1		Model 2	
		All patients	eGFR 10–15	All patients	eGFR 10–15
**12 month analysis**					
Dialysis	eGFR	83 (81–85)^c^	77 (69–87)^c^	82 (80–84)^c^	76 (68–85)^c^
	M/E	113 (91–140)	98 (71–134)	103 (82–128)	81 (57–115)
Death	eGFR	96 (93–98)^c^	108 (91–128)	94 (92–97)^c^	107 (90–127)
	M/E	22 (14–33)^c^	21 (11–40)^c^	26 (16–41)^c^	22 (11–45)^c^
Combined	eGFR	88 (86–89)^c^	85 (78–93)^c^	87 (85–88)^c^	84 (77–93)^c^
	M/E	75 (62–92)^a^	70 (52–93)^a^	75 (61–92)^b^	63 (46–87)^b^
**3 month analysis**					
Dialysis	eGFR	79 (77–82)	79 (67–93)^b^	79 (76–82)^c^	78 (66–92)^b^
	M/E	77 (57–104)	31 (17–53)^c^	70 (52–94)^a^	26 (15–44)^c^
Death	eGFR	96 (93–99)^b^	116 (94–142)	94 (92–97)^c^	114 (92–142)
	M/E	13 (7–22)^c^	9 (4–21)^c^	14 (8–27)^c^	9 (3–23)^c^
Combined	eGFR	87 (85–88)^c^	91 (80–103)	86 (84–88)^c^	91 (80–103)
	M/E	48 (36–63)^c^	20 (13–33)^c^	49 (37–64)^c^	20 (13–33)^c^

^a^:*p* < 0.05; ^b^:*p* < 001;^c^:*p* < 0.001

**Fig. 1:**
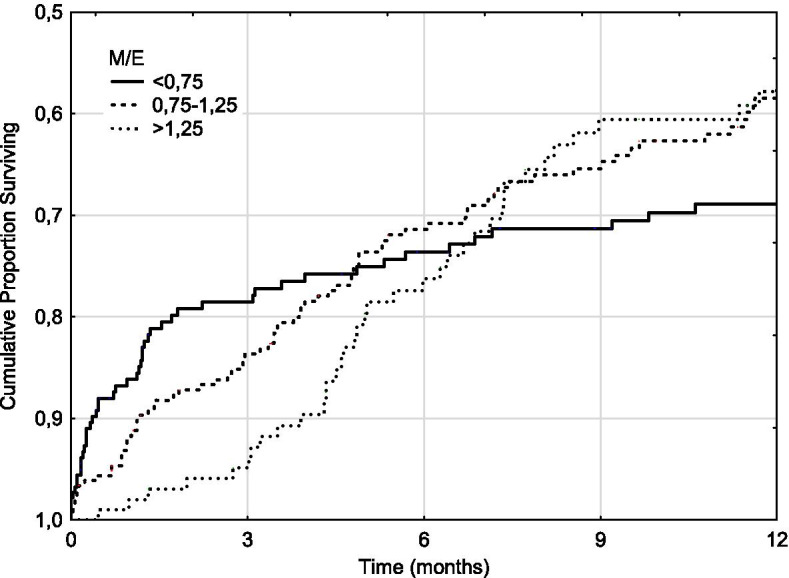
M/E and dialysis incidence. Patients with eGFR 10–15 ml/min/1.73m^2^ only

**Fig. 2:**
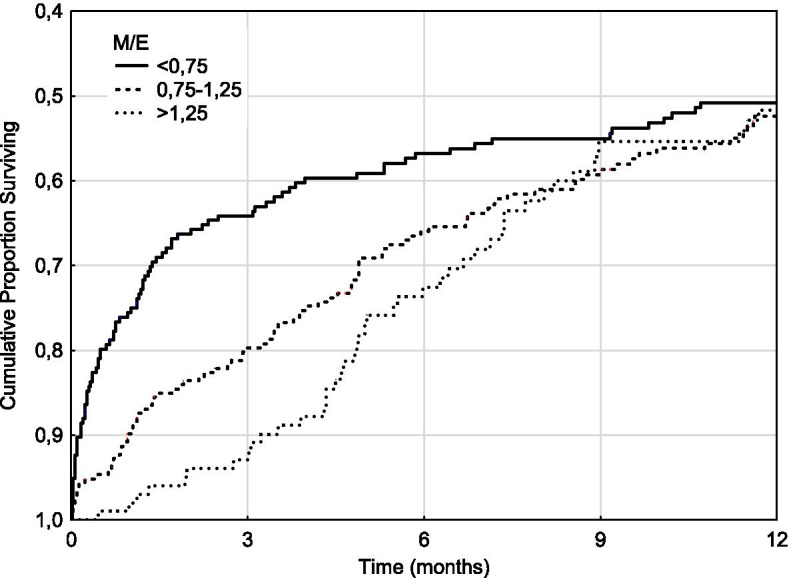
M/E and combined dialysis and mortality incidence. Patients with eGFR 10–15 ml/min/1.73m^2^ only

**Fig. 3:**
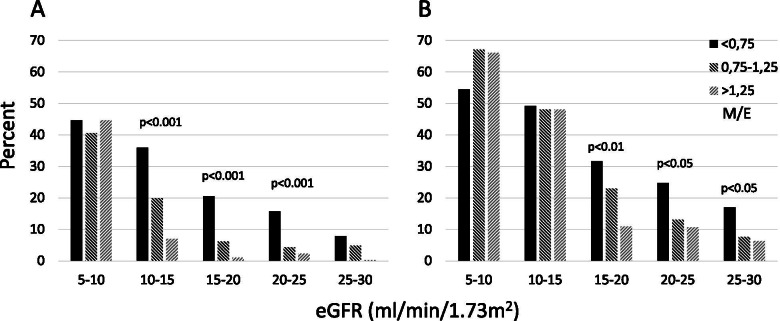
Combined incidence of dialysis and mortality (%) related to M/E and eGFR. A: 3 month; B: 12 month

Perusal of the results seemed to show a dichotomous relationship between M/E and DI at 3 and 12 months. A supplementary analysis showed a significant relationship between M/E and DI for patients with an eGFR of 10–15 ml/min/1.73m^2^ (Figs. 1 and 2). The results were similar for the subgroup of patients with multiple M/E determinations (62.0%), and for patients whose M/E rose between the first and last determination. The results were also similar for all eGFR groups above 10 ml/min/1.73 m^2^ (Fig. 3). Uncensored results are shown in Supplementary Table 2.

Clinical details of patients experiencing planned and unplanned DI are shown in Supplementary Table 1. Categorical analysis of unplanned DI was performed using the following M/E groups: low (< 0.75), medium (0.75–1.25) and high (> 1.25). Unplanned DI was associated with M/E (*p* < 0.001). For patients with final eGFR 10–15 ml/min/1.73m^2^ (114 patients), unplanned DI occurred in low: 44%, medium:29%, high:22%. For patients with eGFR 5–10 ml/min/1.73m^2^ (93 patients) the figures were 51, 40 and 12% respectively.

A supplementary analysis including deaths occurring after DI showed a significant (*p* < 0.001) relationship to mortality 1 year after final M/E measurement (high 6.9%, medium 10.3%, low 23.1%). A subgroup of 195 patients with recent data prior to DI were followed until death or lost-to-follow up. Patients who had a high M/E prior to DI had a significantly lower mortality 1 year after DI and vice versa (high 2.7%, medium 7.3%, low 18.6%, *p* < 0.01). They also had a lower eGFR at DI (high 7.0 (5.1–8.6), medium 8.2 (6.2–10.3), low 9.9 (6.9–13.1), *p* < 0.01).

## Discussion

The M/E was chosen instead of the mGFR for two reasons. mGFR is a somewhat cumbersome investigation for the patient. While eGFR is frequently measured, mGFR measurement will be more uncommon. Assuming that M/E is constant over a period of time and over a range of eGFR, the ratio could be used to estimate the expected mGFR for any proximate eGFR measurement. The first assumption was valid, in that M/E measurements were highly correlated to previous determinations. Both M/E and M/E_BSA_ were negatively correlated to eGFR. There was thus some mathematical coupling between the two variables. This is not surprising, since p-creatinine is included in both equations. Some 4% of the variation in M/E could be attributed to this.

The M/E was highly negatively correlated to a number of comorbidities, malnutrition (as assessed by p-albumin), and inflammation (C-reactive protein). This is not surprising, since sarcopenia, with associated reduced creatinine production, is common to these conditions, which will lead to an increase in eGFR (and a corresponding increase in M/E), independent of any real change in GFR.

The M/E had no predictive value for death or dialysis requirement if the eGFR was < 10 ml/min/1.73m^2^). Above this level, M/E was associated with reduced mortality and combined mortality/DI at 3 and 12 months. In contrast, M/E was only negatively associated with dialysis requirement at 3 but not 12 months. The reason for this difference is unclear. Since the indications for urine collection were unknown, it is possible that these were preferentially made in the presence of acute illness, e.g., related to hospital admission. Furthermore, there were competing risks between DI and mortality. M/E was highly correlated to unplanned DI. These results suggest that patients with a low M/E are at risk of early DI, unplanned DI, and increased mortality. Early dialysis planning for this patient group may reduce the incidence of unplanned DI.

It could be argued that M/E is just a marker of comorbidity, which per se is known to be associated with early requirement of dialysis relative to eGFR [8–14]. However, the relationship to outcomes remained after adjustment for comorbidity. The combined effects of comorbidities are difficult to quantify; M/E could perhaps replace it in assessing requirement for early dialysis.

eGFR at DI was lower in patients with a high M/E, who also had an improved survival after DI. This supports the hypothesis that the paradoxical positive correlation between eGFR at DI and mortality is due to insufficient correction for sarcopenia, and that mGFR is a better measure om the patient’s clinical condition and prognosis. The beneficial relationship of M/E to survival may thus be due to it being a good marker of nutritional status.

M/E was higher in patients treated with ACE inhibitors and diuretics, but not any other class of drugs. Physiological explanations for these associations are unclear, and the lack of relationship to angiotensin 2 antagonist treatment argues against a causal relationship between the renin-angiotensin system activation and M/E. It cannot be argued from these findings that current medical treatment should be considered when assessing the M/E.

This study suffers from several limitations. As it was a retrospective registry study, some data was missing. In particular, BSA was only available in a minority of patients. mGFR and M/E should a priori be normalized for BSA, in order to compare with eGFR. However, the subgroup of patients with BSA measurements did not differ substantially from the group as a whole and the correlations to eGFR were similar. The paucity of BSA data may have skewed the survival analyses. The circumstances and reasons for measuring urea excretion, in addition to the more common creatinine excretion, were not available. If collections were primarily collected in acutely ill patients, then the findings may be less applicable to out-patients. The Cox proportional hazards model does not fully take into account competing hazards (death vs. dialysis); this will have been particularly relevant for patients with eGFR 5–10 ml/min/1.73m^2^. The relationship between DI and M/E was only present on a post hoc analysis of three-month results. eGFR (but not M/E) will probably have played a role in assessing requirement for DI, creating a factitious relationship. The hypothesis thus needs to be validated by an investigation of a population of CKD stage 4 and 5 patients prospectively followed in a specialist clinic.

## Conclusions

In conclusion, a low mGFR/eGFR ratio was associated with comorbidity, malnutrition, inflammation and biochemical uraemia. It was a marker of early DI, death, and unplanned dialysis initiation, independently of eGFR, age, comorbidity and BSA. Particular attention paid to patients with a low M/E may lower the incidence of unplanned dialysis requirement.

## Supplementary Information

**Additional file 1.**

## Data Availability

The dataset supporting the conclusions of this article is available from the corresponding author.

## Abbreviations

ACE: Angiotensin converting enzyme
ANCA: Anti neutrophil cytoplasmic antibody
BSA: Body surface area
CKD5: Chronic kidney disease stage 5
CKD-EPI: Chronic Kidney Disease Epidemiology Collaboration
eGFR: Estimated GFR
eGFR_BSA_: Estimated GFR corrected for BSA
ESA: Erythropoiesis stimulating agents
DI: Dialysis initiation
GFR: Glomerular filtration rate
IQR: Interquartile range
MANOVA: Multiple analysis of variance
M/E: mGFR/eGFR
mGFR: Measured GFR
mGFR_BSA_: Measured GFR corrected for BSA
R: Regression coefficient
SD: Standard deviation
