# Supplementary material for: The ratio of measured to estimated glomerular filtration rate may be a marker of early mortality and dialysis requirement
Source: BMC Nephrol. 2021 Nov 7;22:370. doi: 10.1186/s12882-021-02561-1 (PMC8572425; doi:10.1186/s12882-021-02561-1)
Supplement: Supplementary file 1 — Additional file 1. [file 12882_2021_2561_MOESM1_ESM.docx]

**Supplementary Table 1.** Clinical details of patients experiencing planned and unplanned initiation of dialysis. Percentage in brackets

|  | **Planned** | **Unplanned** |  |
| --- | --- | --- | --- |
| Number | 132 (63) | 76 |  |
| Age (yrs) | 61.2 ±14.2 | 63.8 ±14.2 |  |
| Female | 51 (39) | 31 (41) |  |
|  |  |  |  |
| Renal diagnosis |  |  |  |
| Glomerulonephritis | 34 (26) | 19 (25) |  |
| Chronic interstitial nephropathy | 17 (13) | 7 (9) |  |
| Polycystic disease | 16 (12) | 3 (4) |  |
| Diabetic nephropathy | 29 (22) | 16 (21) |  |
| Hypertensive | 19 (14) | 10 (13) |  |
| Other/unknown | 17 (13) | 21 (28) |  |
|  |  |  |  |
| Previous myocardial infarction | 12 (9) | 8 (11) |  |
| Heart failure | 22 (17) | 12 (16) |  |
| Other heart disease | 72 (55) | 44 (58) |  |
| Cerebrovascular | 18 (14) | 13 (17) |  |
| Peripheral atherosclerosis | 22 (17) | 10 (13) |  |
| Pulmonary disease | 22 (17) | 11 (14) |  |
| Hepatic disease | 3 (2) | 5 (7) |  |
| Cancer | 26 (20) | 9 (12) |  |
| Diabetes | 59 (45) | 34 (45) |  |
|  |  |  |  |
| eGFR at dialysis (ml/min/1.73^2^) | 9.5 ±4.9 | 10.0 ±5.3 |  |
| Last M/E | 1.01 ±0.41 | 0.80 ±0.39^a^ |  |
| Multiple M/E determinations | 68 (52) | 45 (59) |  |
| Time from last M/E to dialysis (months) | 3.1 (1.2-5.2)* | 2.2 (0.05-4.6)^a^ |  |

*: median (interquartile range). ^a^:p<0.001

**Supplementary Table 2.** Relationship of M/E to uncensored dialysis and mortality incidence at 3 and 12 months, classified by eGFR category. Last M/E determination in each category only.

|  |  | **Dialysis** | | | **Mortality** | | |  |
| --- | --- | --- | --- | --- | --- | --- | --- | --- |
| eGFR | Month | M/E | | | M/E | | | |
|  |  | <0.75 | 0.75-1.25 | >1.25 | <0.75 | 0.75-1.25 | >1.25 | |
| 5-10 | 3 | 32 (28) | 49 (38) | 34 (41) | 20 (18) | 4 (3) | 2 (2)^c^ | |
|  | 12 | 38 (34) | 67 (52) | 52 (63)^c^ | 23 (20) | 9 (7) | 2 (2)^c^ | |
| 10-15 | 3 | 36 (20) | 33 (16) | 5 (5)^b^ | 30 (16) | 9 (4) | 2 (2)^c^ | |
|  | 12 | 49 (27) | 77 (37) | 37 (37) | 40 (22) | 16 (8) | 7 (7)^c^ | |
| 15-20 | 3 | 16 (11) | 3 (2) | 0 (0)^c^ | 15 (10) | 9 (5) | 1 (1)^a^ | |
|  | 12 | 23 (15) | 23 (12) | 6 (8) | 24 (16) | 16 (8) | 1 (1)^b^ | |
| 20-25 | 3 | 7 (6) | 3 (2) | 0 (0) | 10 (9) | 4 (3) | 1 (2)^a^ | |
|  | 12 | 12 (11) | 11 (7) | 3 (7) | 14 (13) | 8 (5) | 1 (2)^a^ | |
| 25-30 | 3 | 2 (2) | 3 (2) | 0 (0) | 7 (6) | 4 (3) | 0 (0) | |
|  | 12 | 7 (6) | 4 (3) | 1 (3) | 12 (10) | 6 (4) | 1 (3) | |
| 5-15 | 3 | 55 (23) | 73 (26) | 36 (24) | 42 (17) | 13 (5) | 4 (3)^c^ | |
|  | 12 | 71 (29) | 120 (42) | 79 (52)^c^ | 54 (22) | 25 (3) | 9 (6)^c^ | |
| 15-30 | 3 | 23 (8) | 8 (2) | 0 (0)^c^ | 29 (10) | 15 (4) | 2 (2)^c^ | |
|  | 12 | 36 (12) | 32 (8) | 9 (8) | 44 (15) | 28 (7) | 3 (3)^c^ | |

^a^:p<0.05; ^b^:p<001;^c^:p<0.001 (group analysis)
